# A high-throughput fluorimetric microarray with enhanced fluorescence and suppressed “coffee-ring” effects for the detection of calcium ions in blood

**DOI:** 10.1038/srep38602

**Published:** 2016-12-05

**Authors:** Yanjun Ding, Jiang Ling, Yuchun Qiao, Zhengjian Li, Zongzhao Sun, Jifeng Cai, Yadong Guo, Hua Wang

**Affiliations:** 1 Department of Forensic Science, School of Basic Medical Sciences, Central South University, Changsha 410013, Hunan, China; 2 College of Chemistry and Chemical Engineering, Qufu Normal University, Qufu 273165, P. R. China

## Abstract

A rapid, ultrasensitive, and high-throughput fluorimetric microarray method has been developed using hydrophobic pattern as the microarray substrate and 3-aminopropyltriethoxysilane-coupled carboxylic acid calcium (APS-CCA) as the fluorescent probes for sensing Ca^2+^ ions in blood. The hydrophobic pattern of the developed Ca^2+^ analysis microarray could largely suppress the “coffee-ring” effects to facilitate the better distribution density of testing microspots toward the high-throughput detections, and especially prevent the cross-contamination of the multiple samples between adjacent microspots. Moreover, the use of APS matrix could endow the CCA probe the enhanced environmental stability and fluorescence intensity, which is about 2.3-fold higher than that of free CCA. The interactions between APS-CCA and Ca^2+^ ions were systematically characterized by UV-vis and fluorescence measurements including microscopy imaging. It was demonstrated that the fluorimetric microarray could display the strong capacity of specifically sensing Ca^2+^ ions with the minimal interferences from blood backgrounds. Such an APS-CCA-based fluorimetric microarray can allow for the analysis of Ca^2+^ ions down to 0.0050 mM in blood, promising a highly sensitive and selective detection candidate for Ca^2+^ ions to be applied in the clinical laboratory.

Calcium ions (Ca^2+^), a kind of essential ions with the important physiological activities in human body^1^, have a close relationship with many vital physiological processes involved in the nerve conduction^2^,^3^, muscle contraction^4^, cardiac contraction^5^,^6^, brain function^7^,^8^, enzyme function^9^, endocrine gland secretion of hormones^10^ and other physiology^11^. At the same time, Ca^2+^ ions entering the cells can serve as the special second messengers of electrical signaling^12^, initiating the intracellular events such as secretion^13^, contraction^14^, synaptic transmission^15^,^16^ and gene expression^17^. Ca^2+^ ions could trigger the fertilization process by guiding the cell growth and differentiation as a specific type of cells and regulating the physiology activities. As a result, the changes in Ca^2+^ ions levels in human body have an inseparable relation with the cell function, signal transduction and cell apoptosis, especially the occurrence and development of the specific diseases. Therefore, it is of particular importance to detect the Ca^2+^ ions in human body fluids (such as blood) for the revelation of cellular activities and clinical diagnosis.

Contemporarily, many classic analytical technologies have been excogitated to detect Ca^2+^ ions, such as ion-selective-electrode^18^,^19^, isotope tracer technique^20^, nuclear magnetic resonance method^21^, atomic absorption spectroscopy^22^ and fluorescence detection^23^,^24^. For example, Knowles *et al*. developed an alternative analytical procedure with the silanized microelectrodes for the accurate measurement of Ca^2+^ and Mg^2+^ ions^19^. In 2004, Basset’s group employed myotubes that was derived from the mice to reveal the calcium responses, which were induced by nicotinic acetylcholine receptor stimulation^20^. Also, Akram *et al*. depicted a profile by using atomic absorption spectrophotometer to probe co-existing metals (Ca, Ni, Zn, Cd, Hg, Mn, Fe, Na, Cu, and Mg) in some important herbal plants^22^. Among these detection methods, the fluorimetric methods are widely recognized with the outstanding advantages such as short response time and high sensitivity^25^,^26^,^27^. Still, most of the fluorescence detection methods may encounter with the big-size instruments^21^,^22^, complex operations^18^,^19^,^20^,^21^,^22^, time-consuming^20^,^21^,^22^, low detection throughput, and especially inconvenient for the in-site application, which may have limited their applications at a large scale. Accordingly, developing a new assay for detecting Ca^2+^ ions with high sensitivity, ideal portability, and high throughput is of considerable interest^28^,^29^,^30^.

Recent years have witnessed the rapid development of optical functional materials or fluorescent dyes used as the probes to detect Ca^2+^ ions, most known as calconcarboxylic acid (CCA,1-(2-hydroxy-4-sulfo-1-naphtylazo)-3-naphtolic acid). For example, Mondal^31^ utilized the spectrophotometric method to determine calcium through the chelation between CCA and Ca^2+^ ions by measuring the absorbance changes. Unfortunately, it is still difficult to achieve the high-throughput detection for the medical analysis of Ca^2+^ ions in biological media like blood. Moreover, in modern detection measurements, lots of analysis methods have been developed for the high-throughput detections of targets, of which the typical one is the fluorimetric microarray. Nowadays, the high-throughput fluorimetric technologies have sparked the increasing interests in practical research applications for the analysis of multiple samples^32^,^33^,^34^. For example, the fluorimetric antibody microarrays have been widely developed for the analysis of a large number of biological targets^35^. In order to improvement of the detection throughput of the microarrays or microchips, many efforts have been devoted^36^,^37^. For example, Roy and co-workers built the dense microspots on a high-throughput microchip for the microRNA detection^37^. The hydrophobic patterns were also employed for the design of testing areas to prevent the cross-contamination of the multiple samples between adjacent microspots for the detections of multiple samples^38^. Notably, these microarrays can serve as the detection platforms with micro-size, celerity, and high sensitivity^39^,^40^, and the high-throughput analysis methods for the evaluation of a variety of complex samples at a time^41^. For example, Liu *et al*. designed a photoluminescent array for exploring multiplex metal ions such as Cd^2+^, Mg^2+^, Ag^+^, and Hg^2+^ ions^42^.

Inspired by these pioneering research studies, in this work, we tried to develop a new high-throughput fluorimetric microarray method for Ca^2+^ ions in human body fluids (i.e., blood) by using hydrophobic hexadecyltrimethoxysilane (HDS) to pattern the microarray substrate and 3-aminopropyltriethoxysilane-coupled carboxylic acid calcium (APS-CCA) as the fluorescent Ca^2+^ probe. The fabrication procedure of the APS-CCA fluorimetric microarray was schematically illustrated in Fig. 1. Here, glass slides were first patterned with HDS and then microspotted with APS-CCA to form the hydrophilic testing dots, resulting in a high-throughput optical microarray for the Ca^2+^ detections, where the addition of Ca^2+^ ions could induce the increased fluorescence intensity of APS-CCA. Moreover, the HDS patterns are transparent enough to be tailored for the optical observations and measurements. Importantly, the HDS-patterned hydrophobic surfaces of microarray would suppress the “coffee-ring” effects so as to improve the distribution of APS-CCA microspots on the microarray deposited with the highly uniform density for the high-throughput detections. Also, the HDS-patterned microarray could functionalize with the lotus-like “self-cleaning” for removing the fouling or cross-pollution of the samples between the adjacent APS-CCA microspots. In addition, the APS-CCA droplets on microarray could display the different colors changing from navy to pale yellow depending on the different concentrations of Ca^2+^ ions, so as to expect the naked-eye observations. Subsequently, the application feasibility of the APS-CCA fluorimetric microarray for the detections of Ca^2+^ ions in human body fluids (i.e., blood) was demonstrated.

## Experimental section

### Reagents and Apparatus

Hexadecyltrimethoxysilane (HDS), 3-aminopropyltriethoxysilane (APS), dimethylcarbinol, 1-Ethyl-3-(3-dimethylaminopropyl)carbodiimide hydrochloride (EDC) and N-hydroxysulfosuccinimide sodium salt (NHS) were obtained from Aladdin reagent (Shanghai) co., LTD. Concentrated sulfuric acid, hydrochloric acid and sodium hydroxide were supplied by Lai Yang city tower chemical products factory. Calconcarboxylic acid (CCA) was purchased from Shanghai flute cypress chemical technology co., LTD. A standard Ca^2+^ ions solution was prepared by diluting a calcium chloride (CaCl_2_) aqueous solution (Shanghai flute cypress chemical technology co., LTD.). Blood samples were provided by clinical laboratory of Xiangya hospital. All the other chemicals were of analytical grade and were used as received.

UV-visible absorption spectra were obtained by Lambda 750 spectrophotometer (PerkinElmer, America). Fluorescence spectra were collected on a FluoroMax-4 spectrometer (HORIBA JobinYvon, French). Contact angles were measured by JY-PHb contact angle measuring instrument (Chengde gold co., LTD.) Ultraviolet transilluminator CUV-10 and Inverted fluorescence Microscope (Olympus Optical Co., Ltd. Japan) were used to take fluorescent photos.

### Preparation of fluorimetric microarray

Hydrophobic glasses were prepared in a typical method^43^. An aliquot of 38 ml dimethylcarbinol, 1.1 ml HDS, and 0.16 ml sulfuric acid were mixed in beaker, with the weight percentages of 89%, 10% and 1.0%, respectively. The cleaned glass slides were immersed into the above mixture for about 10 min, and then dried in the fume hood at room temperature. Following that, an aliquot of 1.2 μL of the mixture containing 1.0% APS and 0.50 mM CCA was microspotted onto the slides to be dried overnight, resulting the fluorimetric microarray for future usage.

### The detection applications for Ca^2+^ ions

The selective detections for Ca^2+^ ions in water and real blood were conducted by the following procedure. Firstly, after the preparation of array, a series of 1.0 μL of Ca^2+^ ions solutions with different concentrations from 0.010 mM to 2.0 mM diluted by double-distilled water were separately added onto the microspots of APS-CCA. After 6 min at room temperature to ensure the completed reaction, the changes of fluorescence intensities of the resulting solutions were recorded under the ultraviolet transilluminator. Secondly, the control tests for 2.0 mM metal ions (Zn^2+^, Cd^3+^, Ca^2+^, K^+^, Cu^2+^, Ni^2+^, Fe^2+^, Mg^2+^, Ba^2+^, Al^3+^, Co^2+^, Ag^+^, Pb^2+^, Hg^2+^, Cr^3+^, Na^+^, Mn^2+^ ions) were conducted accordingly. Thirdly, by following the same procedure above, the developed fluorimetric microarray assays were applied to detect Ca^2+^ ions of different concentrations ranging from 0.020 mM to 1.8 mM that were prepared by being separately spiked in the fresh blood samples. Herein, the enhancing efficiencies of APS-CCA by Ca^2+^ ions were calculated according to the relative fluorescence (F − F_0_)/F_0_, where F_0_ and F refer to the fluorescence intensities of APS-CCA (λ_em_ 335 nm) in the absence and presence of Ca^2+^ ions, respectively. Finally, the results of probing Ca^2+^ ions in real blood samples obtained by the developed method were compared with the classic inductively coupled plasma-mass spectrometry (ICP-MS) method used in the clinical laboratory.

## Results and Discussion

### The preparation of fluorimetric microarray

**Figure 1:**
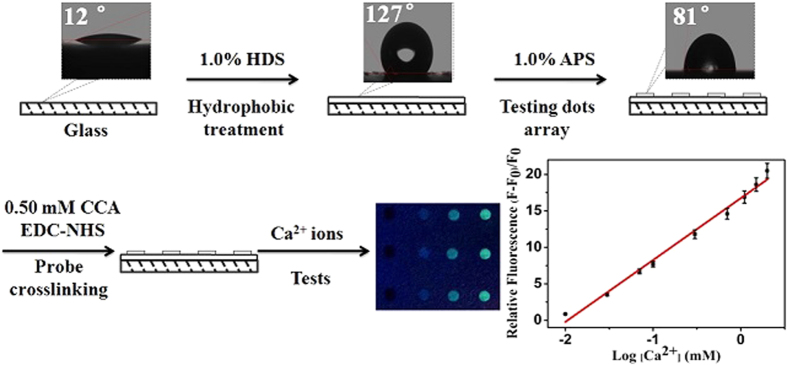
Schematic illustration of the fabrication procedure of the APS-CCA fluorimetric microarray created first with hydrophobic HDS pattern and then with hydrophilic APS-CCA probe microspots.

It is generally recognized that the common detection microarrays can suffer from the low distribution density of testing microspots and the risk of undesirable fouling or cross-contamination of multiple samples, which can be resulted from the “coffee ring” effects of the sensing substances or probes to be immobilized. Moreover, it was well demonstrated that the design of hydrophobic surfaces could be expected to suppress the “coffee-ring” effects^29^,^44^,^45^. Accordingly, a fluorimetric microarray was proposed by first patterning the substrates of glass slides with the hydrophobic HDS and then the immobilization of testing dots of fluorescent APS-CCA probes, with the fabrication procedure schematically illustrated in Fig. 1. One can find that the glass slide could present the contact angle of 12°, which could turn up to 127° once the hydrophobic HDS patterns were formed. When the APS-CCA probes was microspotted onto the HDS patterns, the contact angles could change from 127° to 81°. Accordingly, the introduction of APS matrix could promote the hydrophilicity of the testing microspots, which is of great importance for anchoring the probes for biological molecules. Furthermore, fluorescent CCA was covalently bound onto amine-derivatized APS to obtain the testing dots of APS-CCA, of which the fluorescence intensity could be enhanced after adding Ca^2+^ ions. Also, the so developed Ca^2+^ microarray could exhibit the APS-enhanced environmental stability and fluorescence intensity of CCA. More importantly, the hydrophobic HDS pattern would help to achieve the highly uniform and dense deposition of testing dots on the microarrays for the high-throughput detections, resulting from that the hydrophobic surfaces could influence the evaporation of droplets both in the constant contact radius and contact angle modes towards the large suppression of the “coffee-ring” effects^29^,^44^,^45^, Also, the hydrophobic surfaces might provide a self-cleaning interface between the sample microspots so as to prevent the possible background interference or nonspecific adsorption and the cross contamination from the multiple samples of APS-CCA on the microarrays. Therefore, the so created fluorimetric microarray with the dense and uniform testing dots of APS-CCA probes would be expected for the high-throughput detections of Ca^2+^ ions with the sensitive and reproducible signal outputs.

### Characteristics of APS-CCA probes

**Figure 2:**
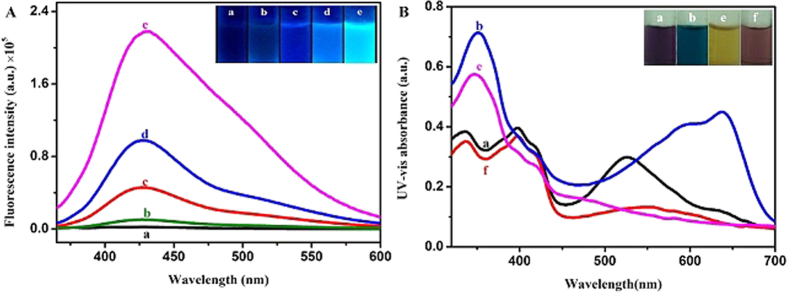
(**A**) Fluorescence intensity spectra and (**B**) UV-vis absorption spectra of (a) 0.50 mM CCA, (b) 1.0% APS with 0.50 mM CCA, (c) 0.50 mM CCA with 0.20 mM Ca^2+^ ions, (d) APS-CCA with 0.20 mM Ca^2+^ ions, (e)APS-CCA with 2.0 mM Ca^2+^ ions, and (f) 0.50 mM CCA with 2.0 mM Ca^2+^ ions.

CCA was selected as the model fluorescent probe for Ca^2+^ ions. The unique optical properties of the obtained APS-CCA were characterized by fluorescence measurements (Fig. 2A). As shown in Fig. 2A, the fluorescence intensity of APS-CCA could itself show a little of fluorescence in double-distilled water, compared to free CCA. After addition of 0.20 mM Ca^2+^ ions, the fluorescence intensity of free CCA increased up to 0.43×10^5^ a.u. In contrast, the fluorescence intensity of APS-CCA could dramatically increase to 0.97×10^5^ a.u., which could be 2.3-fold higher than that of free CCA. It could continue to increase to 2.16×10^5^ a.u. at 2.0 mM Ca^2+^ ions. Obviously, the insert picture in Fig. 2A shows a fluorescence enhancement process. Such a straightforward fluorescence enhancement protocol can circumvent the disadvantage of low response sensitivity of CCA for sensing Ca^2+^ ions. Herein, the fluorescence enhancement of APS-CCA by Ca^2+^ ions is presumably due to the non-radiative electron exchange through effective electron or energy transfer process^46^, resulting from the strong electrostatic interaction and metal-ligand coordination between APS-CCA and Ca^2+^ ions. Based on these findings, therefore, the fluorescence intensity of APS-CCA in the microarray could be enhanced by Ca^2+^ ions selectively and sensitively.

Prior to the microarray preparation, the interactions of Ca^2+^ ions with APS-CCA or CCA were investigated by monitoring the changes of their UV-vis absorption spectra. As shown in Fig. 2B, the maximum absorption of CCA peaks at about 521 nm. For APS-CCA, the absorption peak could be observed at about 641 nm, together with a color changing from purple to navy (Fig. 2B, insert). Interestingly, the UV-vis absorbance of APS-CCA at 641 nm decreased after the exposure to 2.0 mM Ca^2+^ ions, and followed by the color changed to pale yellow. Therefore, the interaction between the APS-CCA and Ca^2+^ ions could cause a dramatic color change, which reflected its fluorescence enhancement process synchronously. By contrast, the CCA without APS turned to light brown in the presence of 2.0 mM Ca^2+^ ions, which color is hard to be distinguished by a naked eye. Moreover, the binding constants for CCA/Ca^2+^ and APS-CCA/Ca^2+^ are calculated as 1.1×10^6^ M^−1^ and 1.7×10^6^ M^−1^, respectively. By comparison, the APS-CCA could apparently present the stronger combining capacity with Ca^2+^ ions than CCA.

**Figure 3:**
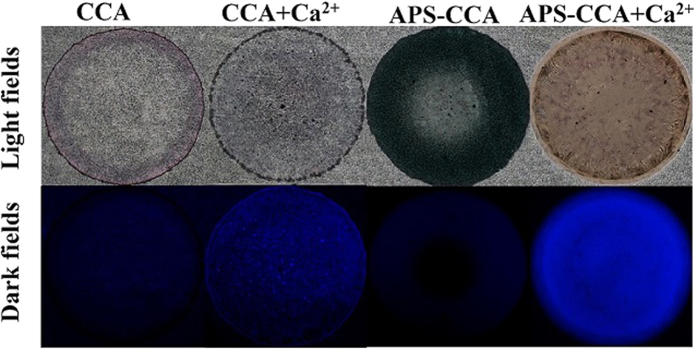
Fluorescent microscope images of CCA and APS-CCA probes in the absence and presence of Ca^2+^ ions (2.0 mM) under the light (up) and dark (down) fields.

Figure 3 shows the investigation for the interaction between APS-CCA and Ca^2+^ ions by using the inverted fluorescence microscope. As characterized by the fluorescent images in light field, CCA could turn to navy after being bound with APS to yield the probe of APS-CCA. The droplet of APS-CCA could become pale yellow after the addition of Ca^2+^ ions, showing an enhanced fluorescence that is about 2.3-fold higher than that of common CCA. Additionally, the images in dark field disclose that APS-CCA or CCA itself could hardly present fluorescence. Moreover, the fluorescence intensity of CCA could be slightly enhanced after the addition of Ca^2+^ ions, indicating that CCA could display a low response to Ca^2+^ ions. However, a dramatically enhanced fluorescence was obtained for APS-CCA after the addition of Ca^2+^ ions. The results indicate that a significant fluorescence enhancement could be expected for the fluorimetric APS-CCA sensor in the presence of Ca^2+^ ions. Again, one can find that these probes of CCA and APS-CCA on bare glass slides manifested obviously the “coffee ring” effects, which might challenge the uniform and dense depositions of the probe microspots on the microarray for the high-throughput detections, and would be suppressed by using the hydrophobic patterns afterwards.

### Optimization of the detection conditions

**Figure 4:**
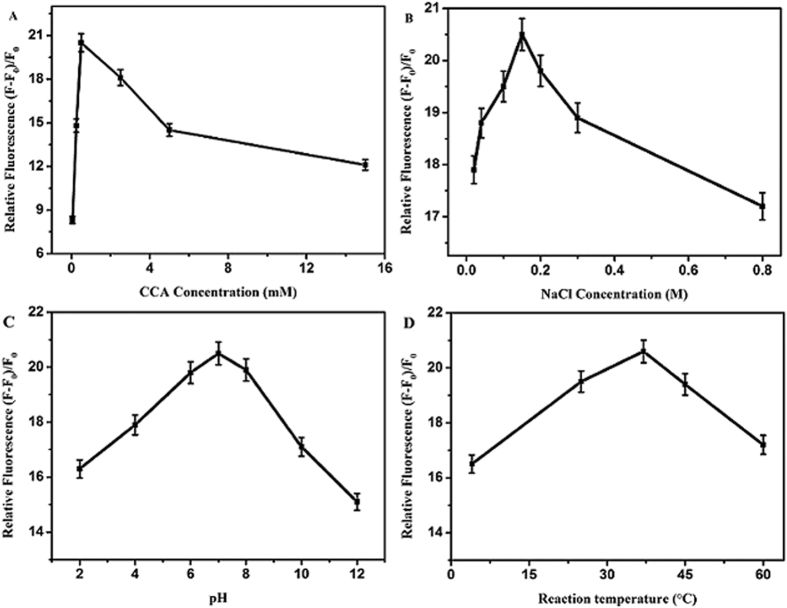
Optimization of the APS-CCA-based fluorimetric conditions of (**A**) CCA concentration-dependent relative fluorescence intensities, (**B**) NaCl concentration-dependent relative fluorescence intensities, (**C**) pH-dependent relative fluorescence intensities, (**D**) reaction temperature-dependent relative fluorescence intensities.

The main conditions for the developed fluorimetric microarray assays were optimized for the detection of Ca^2+^ ions, including the dosages of CCA, pH values, reaction temperature, ions strengths (NaCl concentrations), and reaction time (Fig. 4). Herein, the enhanced efficiencies of APS-CCA caused by Ca^2+^ ions were calculated according to the relative fluorescence intensity of (F–F_0_)/F_0_, where F and F_0_ refer to the fluorescence intensities of APS-CCA (λ_em_ 335 nm) with and without Ca^2+^ ions, respectively. As is shown in Fig. 4A, it was found that the relative fluorescence intensities of APS-CCA increased gradually with the increasing dosages of CCA from 0.050 mM to 0.50 mM, over which they got decreased, indicating that the highest relative fluorescence was obtained at 0.50 mM CCA, which was thus selected as the optimal one. Figure 4B shows the effects of NaCl concentrations on the relative fluorescence intensities of APS-CCA with Ca^2+^ ions. In the presence of Ca^2+^ ions, the relative fluorescence intensities of APS-CCA increased gradually with the increasing NaCl concentrations till 0.15 M, which is close to the concentration of human physiological saline. As the concentrations of NaCl increasing beyond 0.15 M, the relative fluorescence intensities of APS-CCA decreased gradually. Thus, the concentration of NaCl of 0.15 M was chosen for the Ca^2+^ detections. The effects of pH values on the fluorimetric responses of APS-CCA to Ca^2+^ ions were explored with the results shown in Fig. 4C. The relative fluorescence intensities of APS-CCA changed with different pH values, while the addition of Ca^2+^ ions could induce the increase in fluorescence intensities with the different degrees. According to the increased fluorescence efficiencies, pH 7.1 was chosen as the optimal one for the analysis experiments for Ca^2+^ ions. Figure 4D shows the influences of reaction temperature on relative fluorescence of APS-CCA with Ca^2+^ ions. Obviously, the relative fluorescence values could attain the maximum at the reaction temperature of 37 °C.

**Figure 5:**
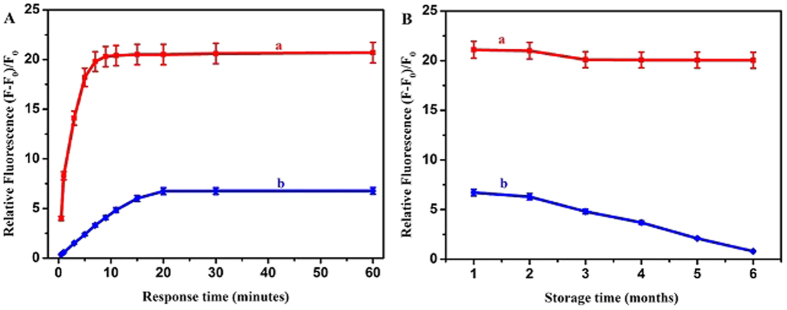
Effects of response time (**A**) and storage time (**B**) on relative fluorescence values of (a) APS-CCA (1.0% APS, 0.50 mM CCA) and (b) 0.50 mM CCA after the addition of Ca^2+^ ions (2.0 mM).

Moreover, the influences of response time on the fluorimetric detections of APS-CCA for Ca^2+^ ions were studied, taking CCA as the comparison (Fig. 5). Obviously, as shown in Fig. 5A, the relative fluorescent intensities of APS-CCA increased drastically upon the addition of Ca^2+^ ions at the first 6 min and then tended to the stationary, indicating the reaction was completed within 6 min. In contrast, the CCA needed 20 min to complete under the same conditions. Also, the ratio of their relative fluorescence values is about 3.1 (20.5/6.7), suggesting a straightforward fluorescence-enhancement protocol was built up. Furthermore, Fig. 5B shows the storage time-dependent fluorescence intensities of APS-CCA and CCA with addition of Ca^2+^ ions separately. As the time changes (1–6 months), the relative fluorescence intensities of the APS-CCA remained about the same. In contrast, the relative fluorescence intensity of CCA was weakened gradually towards being quenched after about 6 months. Such an experimental phenomenon implies that the APS could enhance the fluorescence stability of CCA in the presence of Ca^2+^ ions to promise the significance of practical detection applications.

### The APS-CCA-based fluorimetric analysis for different metal ions

**Figure 6:**
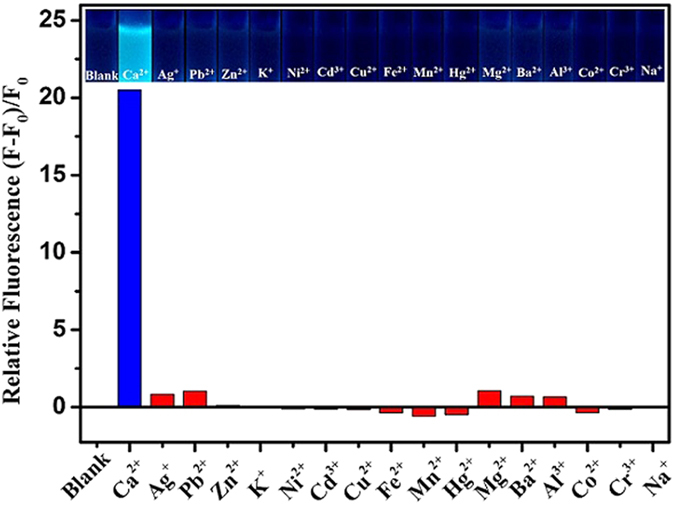
Changes of relative fluorescence intensities of APS-CCA in the presence of different metal ions of 2.0 mM indicated, where F_0_ and F correspond to the fluorescence intensity of APS-CCA in the absence and presence of metal ions, corresponding to the photographs of the solutions (insert).

The APS-CCA fluorimetric microarray was employed for sensing different ions to explore the Ca^2+^ sensing selectivity, including 17 kinds of common metal ions of Zn^2+^, Cd^3+^, Ca^2+^, K^+^, Cu^2+^, Ni^2+^, Fe^2+^, Mg^2+^, Ba^2+^, Al^3+^, Co^2+^, Ag^+^, Pb^2+^, Hg^2+^, Cr^3+^, Na^+^, Mn^2+^ ions (Fig. 6). As expected, the relative fluorescence values of APS-CCA could show the largest response at the addition of Ca^2+^ ions that could trigger the immediate and significant enhancement of the fluorescence of APS-CCA. Accordingly, the APS-CCA could serve as robust fluorescent probes for the selective detections of Ca^2+^ ions. Yet, the addition of Pb^2+^ ions and Mg^2+^ ions could also cause a little of fluorescence enhancement of APS-CCA to some extent, which is neglected compared to Ca^2+^ ions. Such a phenomenon meaningfully confirms that the APS-CCA probe could allow for the specific ability for sensing Ca^2+^ ions.

### Calibration curves for sensing Ca^2+^ ions

**Figure 7:**
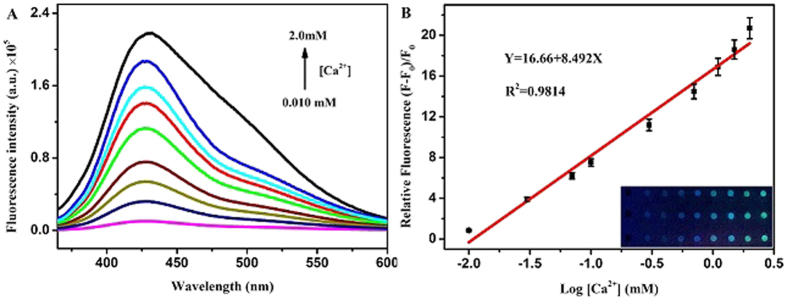
(**A**) Fluorescence intensities of APS-CCA upon the addition of Ca^2+^ ions (0.010–2.0 mM) at λ_em_ 335 nm, (**B**) corresponding to relative fluorescence (F − F_0_)/F_0_ versus the different concentrations of Ca^2+^ ions in water, where F_0_ and F correspond to the fluorescence intensity of APS-CCA in the absence and presence of Ca^2+^ ions, respectively, with corresponding microarray photographs under UV light (insert).

Under the optimal conditions, the developed fluorimetric microarray method was applied for the detection of Ca^2+^ ions. Figure 7A manifests the fluorescent spectra of APS-CCA with Ca^2+^ ions at different concentrations. One can note that the fluorescence intensities increased gradually as Ca^2+^ ions concentrations increased, as also exhibited in the photographs of the corresponding products (Fig. 7B, insert). Figure 7B describes the linear relationship between the concentrations of Ca^2+^ ions *Vs* relative fluorescence intensities. Accordingly, Ca^2+^ ions can be detected over the linear concentrations ranging from 0.010 to 2.0 mM (R^2^ = 0.9814), with a limit of detection (LOD) of 0.0050 mM, which is much lower than the normal value in the human body of about 0.94–1.26 mM^47^. Notably, the fluorescence changes of APS-CCA microspots induced by Ca^2+^ ions of different concentrations could also be observed by naked eyes under ultraviolet transilluminator (Fig. 7B, insert). Moreover, the detection performances of the developed fluorimetric method for calcium ions were compared with those of the other analysis methods^48^,^49^, with the comparison results shown in Table 1. Accordingly, one can note that the developed fluorimetric microarray can display the better or comparable LOD and detection range. Yet, it can possess the additional advantages of high detection throughput towards the simultaneous analysis of multiple samples of calcium ions in the complicated media like blood. Also, the direct and rapid fluorimetric evaluation of the Ca^2+^ levels can be expected by naked eyes under ultraviolet transilluminator.

### Analysis of Ca^2+^ ions in spiked blood samples

**Figure 8:**
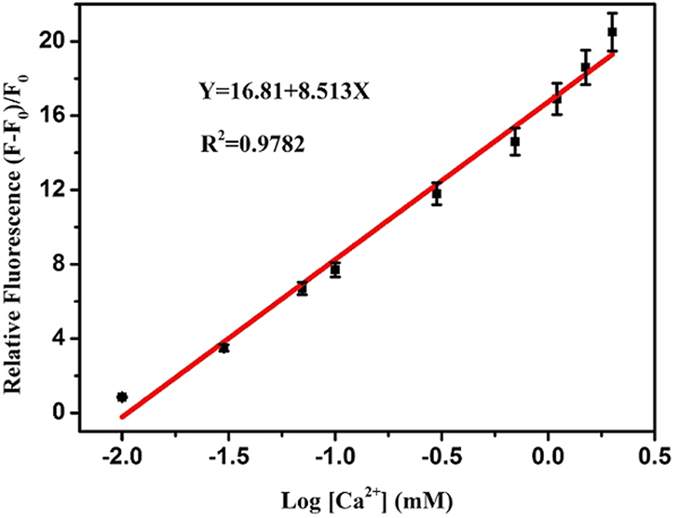
Relative fluorescence intensities (F − F_0_)/F_0_ of APS-CCA versus the different concentrations of Ca^2+^ ions (0.020–1.8 mM) spiked in blood samples.

The application feasibility of the APS-CCA based on fluorimetric microarray method was investigated for Ca^2+^ ions spiked in blood samples with different concentrations. The linear relationship between the increased fluorescence intensity and the concentrations of Ca^2+^ ions was obtained. Accordingly, Ca^2+^ ions in blood could be quantified in the linear concentration ranging from 0.020–1.8 mM (R^2^ = 0.9782), as shown in Fig. 8. Furthermore, the correlation of the analysis results obtained from the developed fluorescent method and the classic ICP-MS method in the clinical laboratory was examined by detecting Ca^2+^ ions in real blood samples. The regression equation for the detection results for Ca^2+^ ions was obtained with correlation coefficient of 0.9751. Obviously, there is no significant difference between the results obtained from the two methods for analyzing Ca^2+^ ions in blood. Therefore, the developed fluorimetric microarray strategy with APS-CCA probe promises the potential feasibility of serving as a rapid and reliable candidate for the selective and sensitive detection of Ca^2+^ ions in blood.

## Conclusions

In summary, a high-throughput fluorimetric microarray has been successfully developed using hydrophobic HDS pattern as the microarray substrate and APS-CCA as the fluorescent probes for sensing Ca^2+^ ions in blood. The so fabricated hydrophobic microarray substrate could suppress the “coffee-ring” effects to facilitate the better distribution density of testing microspots and prevent the cross-contamination of the multiple samples between adjacent microspots toward the high-throughput detections. Moreover, the use of APS could endow the APS-CCA probes the enhanced fluorescence intensity and environmental stability for specifically sensing Ca^2+^ ions. The developed Ca^2+^ fluorimetric microarray was fabricated by the efficient “dip and dry” procedure, without the need for any complicated treatment. The detection results could also be observed by naked eyes if using ultraviolet transilluminator. It should be pointed out that the denser testing microspots would be created on a microarray by a mechanical sample spotter to achieve an even higher throughput of sample analysis. The developed APS-CCA-based fluorimetric microarray strategy is simple, rapid, selective, and highly sensitive, thus holding great promise for the high throughput detection of Ca^2+^ ions in the clinical, food hygiene, and environmental monitoring fields.

## Additional Information

**How to cite this article**: Ding, Y. *et al*. A high-throughput fluorimetric microarray with enhanced fluorescence and suppressed “coffee-ring” effects for the detection of calcium ions in blood. *Sci. Rep.* **6**, 38602; doi: 10.1038/srep38602 (2016).

**Publisher's note:** Springer Nature remains neutral with regard to jurisdictional claims in published maps and institutional affiliations.

## Figures and Tables

**Table 1:** Comparison of Ca^2+^ detection performances among different methods.

Methods	LODs	Detection ranges	Refs
The developed fluorimetry	5.0 μM	10–2.0 × 10^3^ μM	—
The UV-vis measurement	500 μM	500–1.0 × 10^3^ μM	48
The Sol-Gel-based fluorimetry	3.0 μM	3.0–600 μM	49
